# Effect of Die Spacer Thickness on the Microshear Bond Strength of CAD/CAM Lithium Disilicate Veneers

**DOI:** 10.1155/2021/4593131

**Published:** 2021-07-23

**Authors:** Sherine Mohamed Farag, Mona Mohamed Ghoneim, Rania Reda Afifi

**Affiliations:** ^1^Conservative Dentistry Department, Alexandria University, Alexandria, Egypt; ^2^Faculty of Dentistry, Alexandria University, Alexandria, Egypt

## Abstract

**Aim:**

The aim of this study was to compare the microshear bond strength of ceramic veneers with digital die spacer settings at 20, 40, and 100 *µ*m.

**Materials and Methods:**

Eighteen milled lithium disilicate microdiscs (IPS e.max CAD, Ivoclar Vivadent) were divided into three groups (*n* = 6) according to their digital die spacer settings: group A = 20 *µ*m, group B = 40 *µ*m, and group C = 100 *µ*m. Six randomly selected sound maxillary premolars received three microdiscs each. Each microdisc was 1 mm in diameter and 1 mm in height. The buccal surfaces of the premolars were prepared with a 0.5 mm depth in enamel. After cementation, the specimens were thermocycled for 2,500 cycles between 5 and 55°C. Microshear bond strength testing was performed using a universal testing machine until bonding failure. Failure modes were evaluated using a stereomicroscope. Statistical analyses included one-way ANOVA, Tukey's post hoc test, and chi-square test with a 5% alpha error and 80% study power.

**Results:**

The mean microshear bond strength values were calculated in MPa for group A = 31.91 ± 12.41, group B = 29.58 ± 5.03, and group C = 13.85 ± 4.12. One-way ANOVA (*p* ≤ 0.05) showed a statistically significant difference in microshear bond strength among the three groups. Tukey's post hoc test showed significant differences between groups A and C (*p*=0.004) and between groups B and C (*p*=0.011). The failure modes were presented as cohesive, adhesive, and mixed failures. Chi-square test indicated that the failure mode distribution was not significantly different among the three groups (*p*=0.970).

**Conclusion:**

Higher digital die spacer settings decrease the microshear bond strength of CAD/CAM lithium disilicate veneers.

## 1. Introduction

Lithium disilicate ceramic veneers have gained popularity because they combine the properties of strength, longevity, biocompatibility, esthetics, and treatment predictability in a single restoration [1]. Lithium disilicate restorations can be either fabricated using the heat-pressed technique or digitally designed and milled using computer-aided design/computer-aided manufacturing (CAD/CAM) technology [2, 3]. In terms of longevity of the veneer, cementation plays a crucial role [4].

Clearance between the tooth surface and veneer is essential to allow space for the luting resin cement and ensure proper seating and retention. Die spacing is the most common method to provide this space [5]. Die spacing can be defined as the internal relief that allows sufficient and uniform cement thickness for proper seating of an indirect restoration [6]. It can be done manually, by painting on several layers of spacer, or digitally, with CAD technology [7].

Cements with lower viscosities are often assumed to have thinner film thicknesses, since they have greater flow. However, a study by Marcondes et al. showed that this may not always be the case. Some low-viscosity resins may have film thicknesses as high as 119 *µ*m [8]. In this study, no relationships were found between filler content, viscosity, and film thickness. This is because filler content is not the sole factor that determines a resin's properties. Particle type, shape, size, the nature of the particle's surface, and filler spatial arrangement should also be considered. Some previous studies investigated thicknesses ranging from 180 *µ*m up to 500 *µ*m [9, 10]. However, the present study included thicknesses that could be considered within the range of the most common resin film thicknesses.

Previous studies examined the effect of cement thickness on the shear bond strength (SBS) of ceramic veneers and showed that cement thickness and bond strength are inversely proportional. Thinner cement thicknesses produce increased shear bond strengths [11, 12].

Preparations on the wide surface areas of tooth surfaces may not always be even, with some areas having more or less enamel thickness. Microshear specimens have a bonding surface of 1 mm^2^. Such a minuscule surface area on a tooth surface is unlikely to present varying preparation depths. Thus, microshear bond strength (*µ*SBS) testing is more accurate in evaluating bond strength because it allows standard tooth regions to be selected, thus preserving the uniformity of the testing area. Smaller specimens also reduce the possibility of cracks and defects in the test material, as a smaller surface area is involved. In addition, several specimens can be bonded to a single tooth surface [13].

Hence, the present in vitro study was designed to compare different digital die spacer thicknesses on the *µ*SBS of lithium disilicate CAD veneers. The null hypothesis stipulates that various digital die spacer settings will not influence the *µ*SBS of these esthetic restorations.

## 2. Materials and Methods

### 2.1. Tooth Specimen Preparation

The protocol for this research was approved by the Commission of Medical Ethics of Alexandria University under the file number 0124-03/2020.

Six sound freshly extracted maxillary premolars were randomly selected, cleaned of any calculus debris, and stored in a 0.2% thymol solution for 1 week to ensure disinfection. They were then stored in distilled water. Each tooth was marked with a periodontal probe 2 mm below the cementoenamel junction to simulate the periodontal ligament. The roots were then coated with a thin 0.3 mm layer of wax until that mark. After that, they were placed into self-cure acrylic resin in a custom-made copper mold with a diameter of 14 mm and length of 20 mm. The occlusal surface was facing upward, 2 mm below the cementoenamel junction. The teeth and wax were removed from the acrylic blocks, and polyether adhesive (Polyether Adhesive, 3M ESPE, GmbH, Neuss, Germany) was coated on the roots until it dried fully. Polyether impression material was then coated on the roots, and the tooth was returned to its acrylic block to mimic periodontal ligament. The excess impression material (Impregum Soft, 3M ESPE, GmbH, Neuss, Germany) was removed using a sharp scalpel [14].

### 2.2. Tooth Surface Preparation

The six specimens were prepared at a depth of 0.5 mm into the enamel of the buccal surface with a high-speed handpiece and depth limiting and tapered diamond burs of medium grit (Microdont, Monsey, NY, USA) [14]. A stereomicroscope was used to verify that the entire preparation was in enamel.

**Figure 1:**
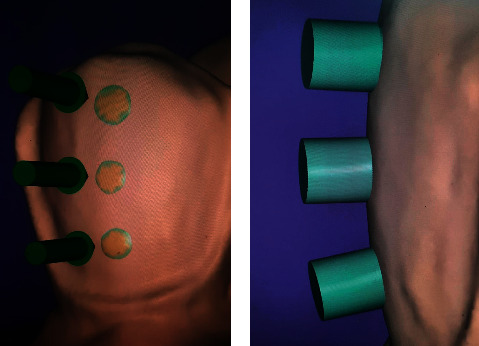
(a) Design of digital die spacer on the buccal surface. (b) Design of microdisc on the buccal surface.

The specimens were scanned with an extraoral scanner (Vinyl scanner, Smart optics Sensortechnik, GmbH, Bochum, Germany). Exocad software (Exocad GmbH, Darmstadt, Germany) was used to individually design the ceramic microdiscs (IPS e.max CAD, Ivoclar Vivadent, Schaan, Liechtenstein) for each specimen. Each microdisc had a diameter of 1 mm and a height of 1 mm. Various thicknesses of die spacer settings were assigned to each group: group A = 20 *µ*m, group B = 40 *µ*m, and group C = 100 *µ*m. Blocks were then milled using the CEREC inLab MC XL CAD/CAM milling machine (Sirona Dental Systems, GmbH, Bensheim, Germany) [9] (Figure 1).

### 2.3. Surface Treatment of Ceramic Microdiscs

The intaglio surfaces of the microdiscs were etched using 8% hydrofluoric acid (HF) (Dentobond etching gel, Itena, Villepinte, France) for 60 seconds, rinsed and air-dried. Silane bifunctional agent (RelyX ceramic primer, 3M ESPE, St. Paul, MN, USA) was applied using a microbrush and allowed to react for 60 seconds and then air-dried for 2–5 seconds [15]. Next, a thin layer of adhesive was coated on the microdiscs and gently dispersed using an air syringe for 5 seconds. A digital light microscope (Inskam 307, Shenzhen, China) was used to view the intaglio surface of the microdisc.

### 2.4. Surface Treatment of Prepared Enamel Surface

**Figure 2:**
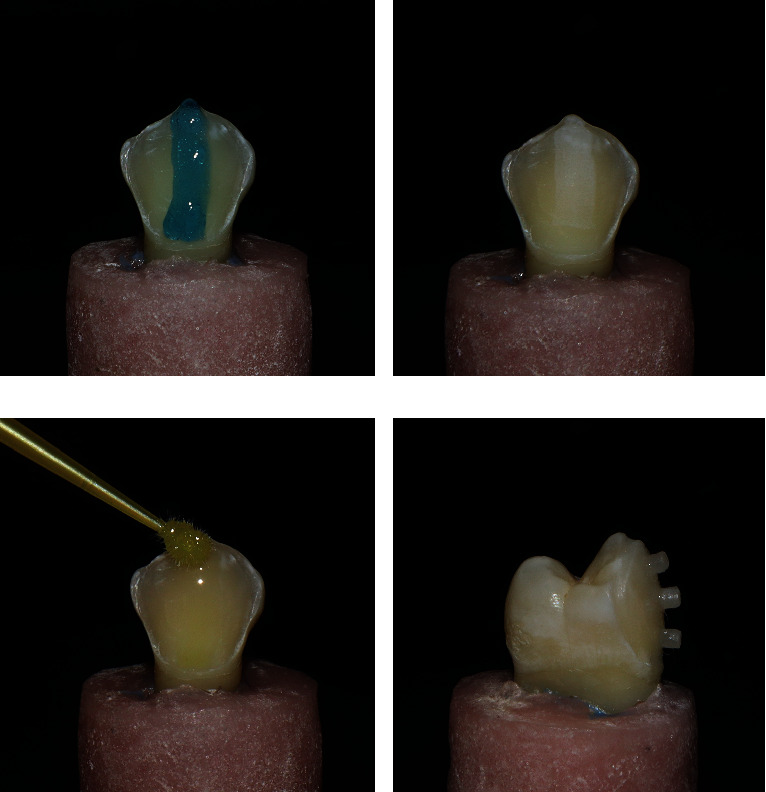
(a) Strip of etching gel at center of buccal surface to prevent the waste of material. (b) Chalky-white surface after rinsing of the etching gel. (c) Application of adhesive. (d) Cementation of microdiscs, proximal view.

To avoid the waste of material, only a thin strip at the center of the buccal surface was etched using 37% phosphoric acid (N-etch gel, Ivoclar Vivadent, Schaan, Liechtenstein) for 15 seconds and then washed thoroughly with water for 10 seconds, and excess water was gently blotted away. Two consecutive coats of universal adhesive (single-bond universal adhesive, 3M ESPE, St. Paul, MN, USA) were applied using a microbrush onto the etched enamel surfaces and agitated for 20 seconds. The adhesive was then dispersed using oil- and water-free compressed air until a glossy, and uniform layer was formed [14] (Figure 2).

### 2.5. Cementation of Microdiscs

A thin layer of resin cement (RelyX Veneer, 3M ESPE, St. Paul, MN, USA) was applied on the area of the tooth, where the microdiscs were then placed. The microdiscs were cemented on three different areas of the buccal surface: occlusal third, middle third, and cervical third. The microdiscs were placed on the tooth surface using a tweezer. Excess cement was removed using a microbrush. They were then light cured for 40 seconds using light-emitting diode unit with a light intensity of 1200 mW/cm^2^ (Elipar™ FreeLight 2, 3M ESPE, St. Paul, MN, USA). All specimens were aged by thermocycling for 2,500 cycles in water baths with a temperature range between 5 and 55°C with a dwell time of 15 seconds in each bath and 5 seconds transfer time [15] (Figure 2).

### 2.6. Microshear Bond Strength Test

**Figure 3:**
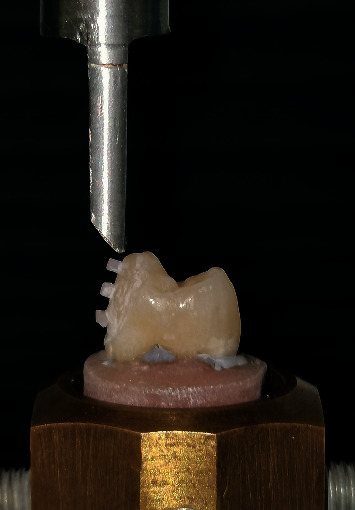
Microshear bond strength test using universal testing machine.

A universal testing machine (5ST Tinius Olsen, Redhill, UK) was used to measure the *μ*SBS of the specimens. A mono-beveled chisel fell at the tooth and restoration interface at a cross-head speed of 0.5 mm/min. The *μ*SBS was expressed in MPa, as derived from dividing the imposed force (N) at the time of fracture by the bonded area (mm^2^) [15] (Figure 3).

### 2.7. Failure Mode Assessment

**Figure 4:**
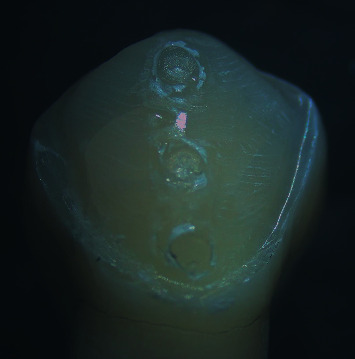
Stereomicroscopic evaluation of failure modes at 18x magnification. Types of failure modes: cohesive (occlusal third), mixed (middle third), and adhesive (cervical third).

The tooth surface was then viewed through a stereomicroscope (SZ114STR, Olympus, Tokyo, Japan) at a magnification of 18x to determine the mode of failure. Failure modes were described as adhesive, cohesive, or mixed. Failures were classified as cohesive if more than 75% of luting resin remained on the tooth surface, adhesive if less than 25% of the luting resin remained on the tooth surface, or mixed if the remaining luting resin was between 25% and 75% [16] (Figure 4).

## 3. Statistical Analysis

The sample size was estimated assuming a 5% alpha error and 80% study power. Based on previous studies, the mean µSBS values were 6.8 MPa [11], 14.19 MPa, and 6.22 MPa [12] for the 20, 40, and 100 *µ*m die spacer groups, respectively. Based on the difference between independent means using F test and a standard deviation (SD) of 2.1 [11], the minimum sample size was calculated to be 5 specimens per group, which was increased to 6 specimens to make up for processing errors. The sample size was calculated using G^*∗*^ power 3.0.10. software (Franz Faul, University of Kiel, Kiel, Germany).

## 4. Results

**Figure 5:**
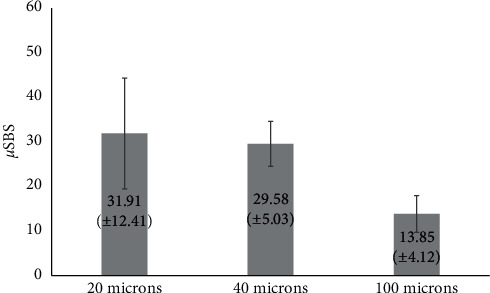
Mean microshear bond strength (MPa) and standard deviations (SD) among the study groups. *p* value = 0.003^*∗*^ (^*∗*^statistically significant difference at *p* value ≤0.05).

The mean values and standard deviations of the µSBS test are shown in Figure 5. One-way ANOVA (*p* ≤ 0.05) indicated a statistically significant difference among the three groups. Tukey's post hoc test showed significant differences between groups A and C and between groups B and C (Table 1). No significant difference was found between groups A and B. There was no significant difference in the failure mode distribution among the three groups (Table 2).

## 5. Discussion

Although there exists a plethora of studies on the shear bond strength of laminate veneers with regard to cement type, ceramic type, ceramic surface treatment, and preparation type [2, 17–19], the role of cement thickness seems to have been less focused on. This study served to help provide this information. The microdiscs in this experiment were chosen to provide precise measurements and avoid an uneven intaglio surface that may happen during the milling of restorations, as mentioned in a study by Venturini et al. [20], where the resulting cement thickness was not identical to the digital die spacer settings of CAD/CAM leucite ceramic crowns. In addition, the cement space thickness was irregular within the same restoration, differing between the cusps and central fossa. A 1 mm bonded surface prevents such discrepancies.

The results of this study show that cement space and bond strength are inversely proportional, as supported in previous studies [11, 12]. As the thickness of cement decreases, bond strength increases. Thus, the null hypothesis was rejected. Cho et al. [11] compared the SBS of porcelain veneers with different numbers of coats of paint-on die spacer. The results showed that groups with two coats of die spacer (12.8 ± 2.62 *µ*m) exhibited higher SBS values than groups with no coats, whereas four coats (26.80 ± 3.90 *µ*m) and six coats (38.09 ± 4.26 *µ*m) of die spacer resulted in similar SBS values. Thermocycling greatly influenced the results of the previous study, which reported a decrease of 42.5% in SBS between the noncoated and coated specimens after 2,500 cycles. Thermocycling can induce ceramic cracks and breakdown the resin cement-enamel bond, which would adversely affect the SBS. Magne et al. found that the damage to ceramic was most extensive when the ratio of ceramic thickness to luting cement was small, which consequently contributed to bond failure [21].

Sabarinathan et al. [12] conducted a similar study where the SBS of porcelain veneers was examined after no coats, two coats (40.55 ± 12 *µ*m), four coats (79.15 ± 32 *µ*m), and six coats (126 ± 58 *µ*m) were applied. The results indicated that the SBS peaked at a die spacer thickness of 40.55 ± 12 *µ*m and decreased as the thickness increased. Those results are within the recommended American Dental Association's range between 25 and 40 *µ*m [22].

The authors attributed this inversely proportional phenomenon between cement thickness and bond strength to the presence of a thick resin margin that may initiate crack propagation within the luting resin, leading to failure within the resin and debonding of the veneer rather than failure at the tooth-resin interface. In the present study, however, there was no significant difference in the overall distribution of failure type. It must be noted that differences in ceramic type, bonding agents, fabrication technique, ceramic surface treatment, number of thermocycling cycles, contamination of the tooth or ceramic surface, and/or light curing intensity may have influenced the distributions of failure types between the current and previous studies. Also, the failure mode for the same type of cement may vary among different studies [2, 23, 24]. As in the studies of Öztürk et al. [2] and Prieto et al. [23], RelyX Veneer cement had a majority of adhesive failures, whereas the same cement in another study produced mostly mixed failures [24].

The mechanism of failure was explained by Liu et al. [9] in a finite element analysis of ceramic veneers with various cement thicknesses. The maximum stresses were at the resin tag base of the enamel-adhesive interface on the lingual side. Bonding failure was found when the micromodels had cement layers higher than 50 *µ*m. Nevertheless, the adhesive stresses on the labial aspect of the veneer were much lower and uniform than those on the lingual aspect. Since the present study consisted of specimens cemented on the labial surface of the tooth, it seems more appropriate to give more consideration to the values of adhesive stress distribution on the labial surface. Failures in ceramic due to shear stress can also be attributed to the elastic modulus of the resin cement (6,000 MPa) being less than that of the ceramic veneer (70,000 MPa) [9]. When a ceramic specimen is uniformly bonded to a less stiff resin cement, high tensile stress is developed in the ceramic at the ceramic-cement interface right below the load. Therefore, thicker cements could lead to ceramic failures [25].

Moreover, volumetric polymerization shrinkage may have played a role in the failures attributed to higher cement thicknesses. A study by Ishikiriama et al. demonstrated increased polymerization shrinkage forces in thicker resin cements. Such forces would have caused tensile stress on the ceramic during cement contraction, initiating cracks [26].

To make the results of this study comparable to those of previous studies, similar methods were used. The enamel surfaces of the current study were etched at 15 seconds and thermocycled for 2,500 cycles, as were the specimens of the two main studies that examined the effect of cement thickness on the SBS of ceramic veneers [11, 12]. Although one study found that enamel bonding was stronger at 30 seconds of acid-etching [27] and that 2,500 cycles may be a short amount of time clinically, the present parameters were necessary to make the results of this study comparable to previous ones.

The *µ*SBS test holds the advantage that its specimens are considerably smaller than those used in the macroshear test. Specimens' bonding surfaces are only 1 mm^2^ and, therefore, are less likely to contain defects that may interfere with the results, as is the case with the macroshear bond test. However, shear bond testing is a widely criticized test, and some limitations of the test hold true for both macro- and microshear bond strength tests. Shear tests tend to bend specimens, exposing the adhesive surface to mainly tensile and compressive forces, instead of subjecting them purely to shear stresses. Perpendicular alignment of the specimens with the chisel was also not possible due to the convexity of the buccal surface. For this reason, the mechanical load at different slopes may have been responsible for the large standard deviation observed in the results [28].

The use of 8% HF instead of 5% concentration for the conditioning of the internal surfaces of the lithium disilicate might be considered a limitation. Some studies reported greater erosion without significant gain of bond strength when HF was increased from 5% to 7.5% [29, 30].

It can be inferred from this study that thicker cements increase stresses in both the tooth-adhesive and cement-ceramic interfaces, causing failure. More investigation should be carried out to provide greater knowledge on the subject.

## 6. Conclusion

Within the limitations of this study, it can be concluded that digital die spacer thickness significantly affects the *µ*SBS of CAD/CAM lithium disilicate. The optimum spacer thickness should be between 20 and 40 *µ*m.

## Figures and Tables

**Table 1:** Post hoc comparisons of study groups regarding *µ*SBS.

Group	Compared to	*p* value
20 microns	40 microns	0.873
	100 microns	0.004^*∗*^

40 microns	100 microns	0.011^*∗*^

^*∗*^Statistically significant difference at *p* value ≤0.05.

**Table 2:** Distribution of failure mode in *µ*SBS among the study groups.

	20 microns (*n* = 6)	40 microns (*n* = 6)	100 microns (*n* = 6)	Chi-square (*p* value)
Cohesive	2 (33.3%)	3 (50%)	2 (33.3%)	*X* ^2^ = 0.536 (0.970)
Adhesive	1 (16.7%)	1 (16.7%)	1 (16.7%)	
Mixed	3 (50%)	2 (33.3%)	3 (50%)	

## Data Availability

The data used to support the findings of this study are available from the corresponding author upon request.

## References

[1] Radz G. M. (2011). Minimum thickness anterior porcelain restorations. *Dental Clinics of North America*.

[2] Öztürk E., Bolay Ş., Hickel R., Ilie N. (2013). Shear bond strength of porcelain laminate veneers to enamel, dentine and enamel-dentine complex bonded with different adhesive luting systems. *Journal of Dentistry*.

[3] Levin G. G., Vishnyakov G. N., Loshchilov K. E., Ibragimov T. I., Lebedenko I. Y., Tsalikova N. A. (2010). Modern dental CAD/CAM systems with intraoral 3D profilometers. *Measurement Techniques*.

[4] Seymour K. G., Cherukara G. P., Samarawickrama D. Y. (2001). Stresses within porcelain veneers and the composite lute using different preparation designs. *Journal of Prosthodontics*.

[5] Tuntiprawon M., Wilson P. R. (1995). The effect of cement thickness on the fracture strength of all-ceramic crowns. *Australian Dental Journal*.

[6] Mule S. A., Dange S. P., Khalikar A. N., Vaidya S. P. (2014). Effect of varying layers of two die spacers on precementation space of full coverage restorations. *The Journal of Indian Prosthodontic Society*.

[7] Hoang L. N., Thompson G. A., Cho S.-H., Berzins D. W., Ahn K. W. (2015). Die spacer thickness reproduction for central incisor crown fabrication with combined computer-aided design and 3D printing technology: an in vitro study. *The Journal of Prosthetic Dentistry*.

[8] Marcondes R. L., Lima V. P., Barbon F. J. (2020). Viscosity and thermal kinetics of 10 preheated restorative resin composites and effect of ultrasound energy on film thickness. *Dental Materials*.

[9] Liu H.-L., Lin C.-L., Sun M.-T., Chang Y.-H. (2009). Numerical investigation of macro- and micro-mechanics of a ceramic veneer bonded with various cement thicknesses using the typical and submodeling finite element approaches. *Journal of Dentistry*.

[10] May L. G., Kelly J. R., Bottino M. A., Hill T. (2012). Effects of cement thickness and bonding on the failure loads of CAD/CAM ceramic crowns: multi-physics FEA modeling and monotonic testing. *Dental Materials*.

[11] Cho S.-H., Chang W.-G., Lim B.-S., Lee Y.-K. (2006). Effect of die spacer thickness on shear bond strength of porcelain laminate veneers. *The Journal of Prosthetic Dentistry*.

[12] Sabarinathan S., Sreelal T., Rajambigai A. A. S. (2016). Evaluation of influence of die spacer thickness on the shear bond strength of porcelain laminate veneers: an in-vitro study. *Indian journal of stomatology*.

[13] Upadhyaya V., Arora A., Singhal J., Kapur S., Sehgal M. (2019). Comparative analysis of shear bond strength of lithium disilicate samples cemented using different resin cement systems: an in vitro study. *Journal of Indian Prosthodontic Society*.

[14] Linhares L. A., Pottmaier L. F., Lopes G. C. (2018). Fracture resistance of veneers in premolars. *European Journal of Dentistry*.

[15] David J., De Matos M., Jiro L., Nakano N., Nadal L., Bottino M. A. (2020). Influence of acid etching on bond strength between feldspathic ceramics and resin cement. *Revista Brasileira de Odontologia*.

[16] Toman M., Cal E., Türkün M., Ertuğrul F. (2008). Bond strength of glass-ceramics on the fluorosed enamel surfaces. *Journal of Dentistry*.

[17] Moses A., Ganesan L., Shankar S., Hariharan A. (2020). A comparative evaluation of shear bond strength between feldspathic porcelain and lithium di silicate ceramic layered to a zirconia core- an in vitro study. *Journal of Clinical and Experimental Dentistry*.

[18] Mokhtarpour F., Alaghehmand H., Khafri S. (2017). Effect of hydrofluoric acid surface treatments on micro-shear bond strength of CAD/CAM ceramics. *Electronic Physician*.

[19] Alavi A. A., Behroozi Z., Nik Eghbal F. (2017). The shear bond strength of porcelain laminate to prepared and unprepared anterior teeth. *Journal of Dentistry (Shiraz, Iran)*.

[20] Venturini A. B., Wandschera V. F., Marchionatti A. M. E. (2020). Effect of resin cement space on the fatigue behavior of bonded CAD/CAM leucite ceramic crowns. *Journal of the Mechanical Behavior of Biomedical Materials*.

[21] Magne P., Kwon K.-R., Belser U. C., Hodges J. S., Douglas W. H. (1999). Crack propensity of porcelain laminate veneers: a simulated operatory evaluation. *The Journal of Prosthetic Dentistry*.

[22] Langham S., Simon J. F., Tantbirojn D., Redmond D., Langham S. (2017). The importance of the cement spacer for proper crown seating. *International Journal of Computerized Dentistry*.

[23] Prieto L. T., de Araújo C. T., Pierote J. J. (2020). Chemical and physical evaluation of the luting systems for veneers submitted to accelerated artificial aging. *Journal of International Dental and Medical Research*.

[24] Alkhurays M., Alqahtani F. (2019). Influence of different luting cements on the shear bond strength of pretreated lithium disilicate materials. *The Journal of Contemporary Dental Practice*.

[25] Karntiang P., Leevailoj C. (2014). Effect of resin cement thickness on compressive fracture resistance of enamel-bonded ceramic. *Cumhuriyet Dental Journal*.

[26] Ishikiriama S. K., Maenosono R. M., Oda D. F., Ordóñez-Aguilera J. F., Wang L., Mondelli R. F. L. (2013). Influence of volume and activation mode on polymerization shrinkage forces of resin cements. *Brazilian Dental Journal*.

[27] Gardner A., Hobson R. (2001). Variations in acid-etch patterns with different acids and etch times. *American Journal of Orthodontics and Dentofacial Orthopedics*.

[28] Armstrong S., Geraldeli S., Maia R., Raposo L. H. A., Soares C. J., Yamagawa J. (2010). Adhesion to tooth structure: a critical review of “micro” bond strength test methods. *Dental Materials*.

[29] Sundfeld Neto D., Naves L., Costa A. (2015). The effect of hydrofluoric acid concentration on the bond strength and morphology of the surface and interface of glass ceramics to a resin cement. *Operative Dentistry*.

[30] Puppin-Rontani J., Sundfeld D., Costa A. (2017). Effect of hydrofluoric acid concentration and etching time on bond strength to lithium disilicate glass ceramic. *Operative Dentistry*.

